# Supplementary material for: The structure, function and evolution of a complete human chromosome 8
Source: Nature. 2021 Apr 7;593(7857):101–7. doi: 10.1038/s41586-021-03420-7 (PMC8099727; doi:10.1038/s41586-021-03420-7)
Supplement: Supplementary file 1 — This file contains Supplementary Figures 1-11, Supplementary Tables 3-5, 7 and Supplementary References. [file 41586_2021_3420_MOESM1_ESM.pdf]

nature portfolio

https://doi.org/10.1038/s41586-021-03420-7

Supplementary information

The structure, function and evolution of a complete human chromosome 8

In the format provided by the authors and unedited

SUPPLEMENTARY INFORMATION FOR:

The structure, function, and evolution of a complete human chromosome 8

This PDF file includes:

1. **Supplementary Figures 1 to 11**
2. **Supplementary Tables 1 to 10**
3. **Supplementary References**

SUPPLEMENTARY FIGURES

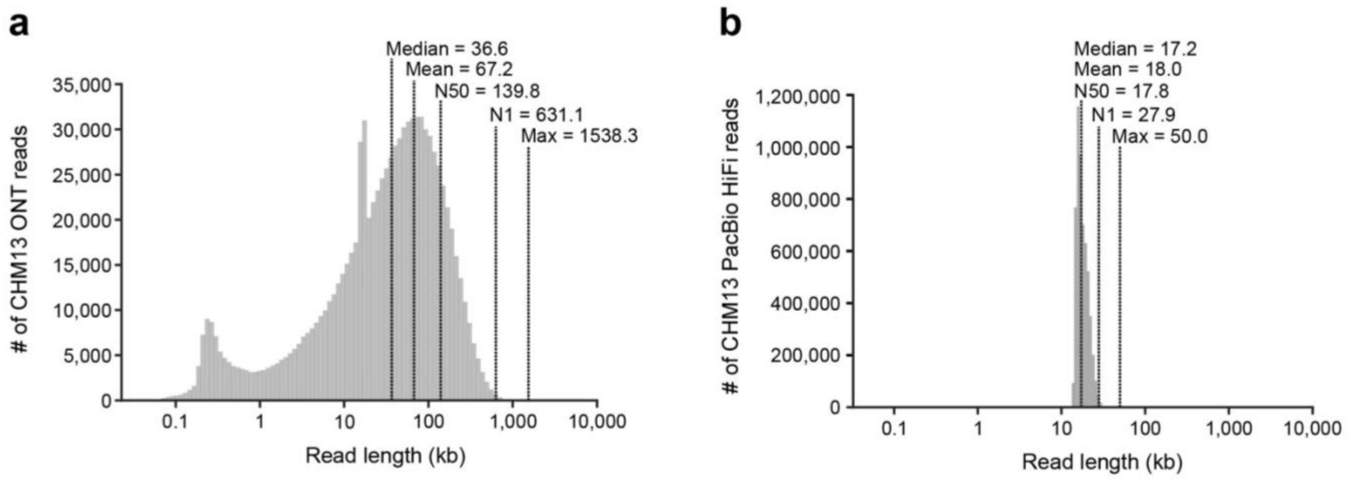

Supplementary Figure 1. Ultra-long ONT and PacBio HiFi data generated from the CHM13 genome. a,b) Read-length distributions of **a)** ultra-long ONT and **b)** PacBio HiFi data generated from the CHM13 genome for this study. The median, mean, N50, N1, and max read lengths are indicated.

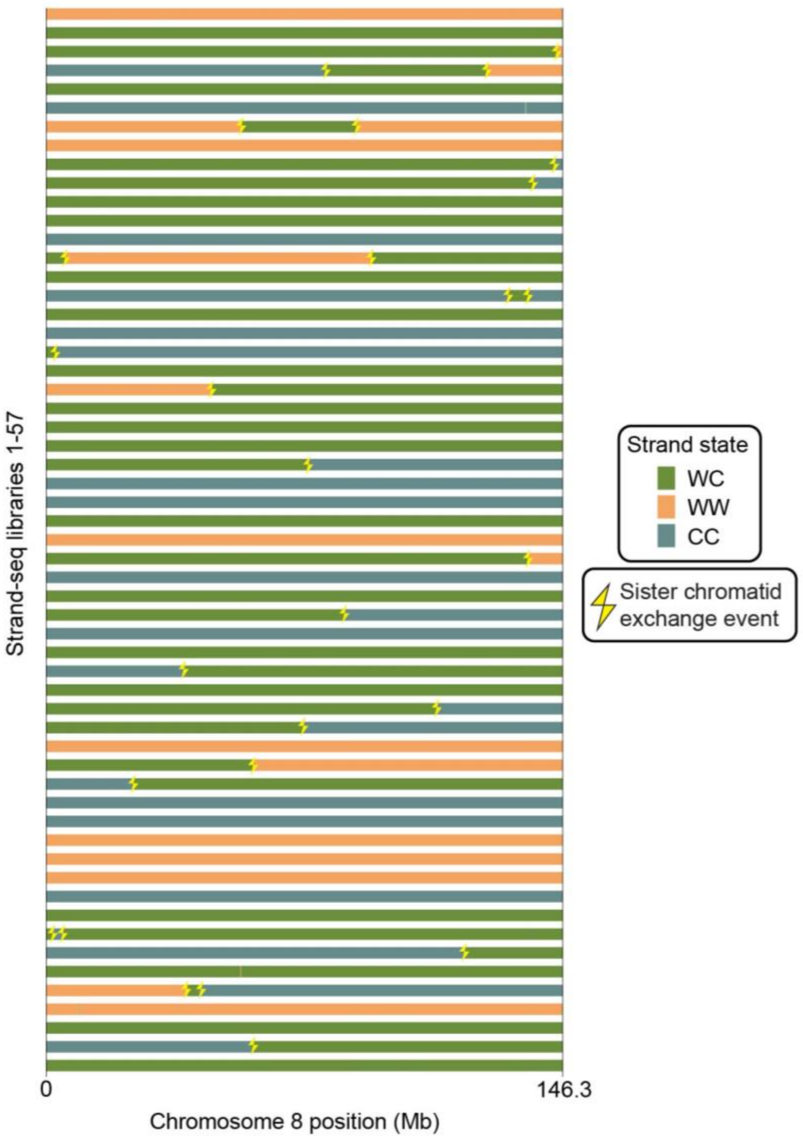

Supplementary Figure 2. Strand-seq validation of the CHM13 chromosome 8 assembly. Strand-seq is a single-cell sequencing technique able to assess directional and structural contiguity of individual homologs by sequencing only template single-stranded DNA[1–3]. Horizontal colored bars show Strand-seq strand states for 57 libraries. Such strand state changes are normally caused by a double-strand-break that occurred during DNA replication and was repaired by a sister chromatid[3]. Observed strand state changes are randomly distributed along each Strand-seq library (yellow thunderbolts) for chromosome 8 and, thus, are not indicative of a genome misassembly. Genome misassemblies are indicated by a recurrent change in strand state over the same region in multiple Strand-seq cells[4]. WC: Watson-Crick; WW: Watson-Watson; CC: Crick-Crick.

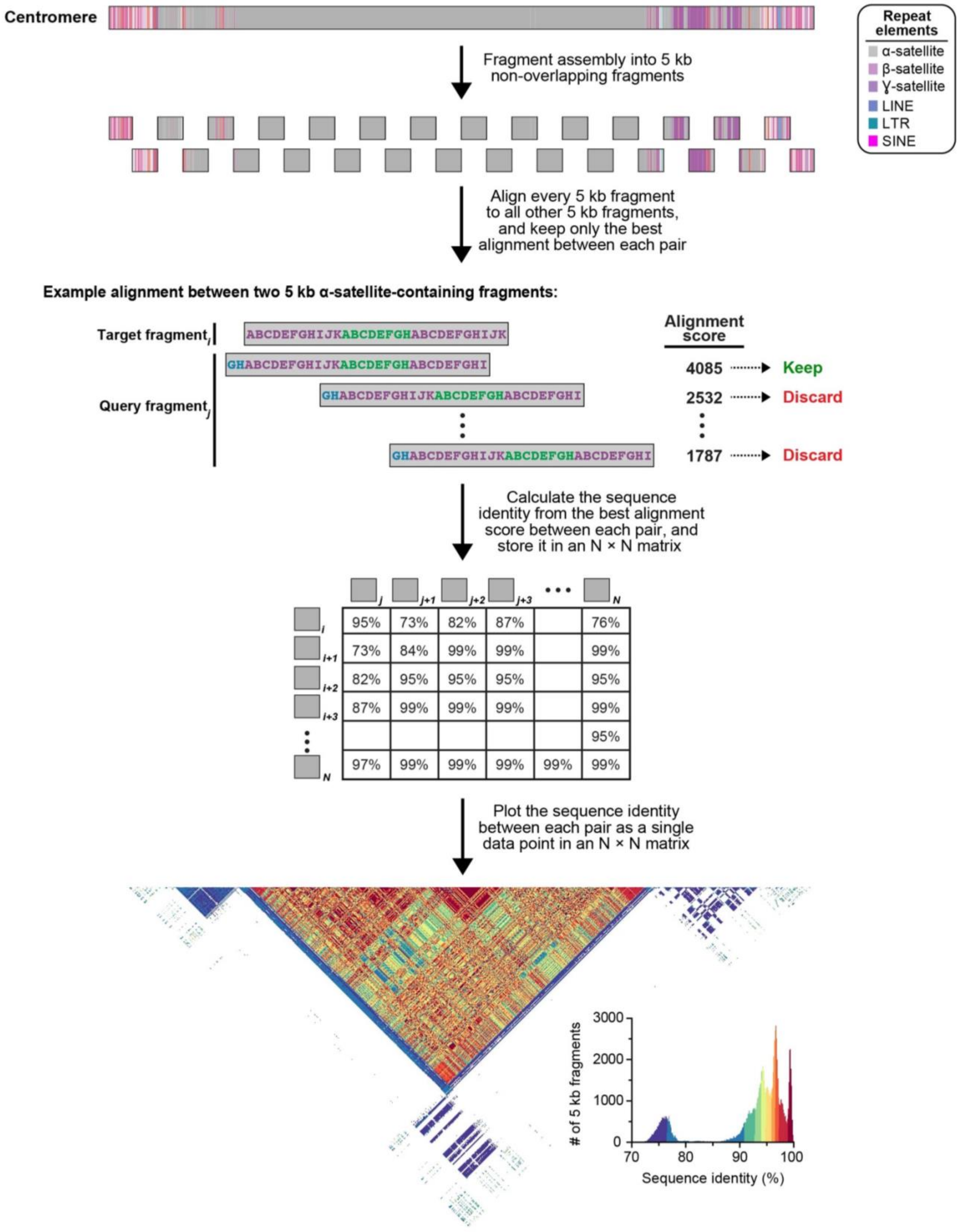

Supplementary Figure 3. Process to generate a pairwise sequence identity heatmap. To generate a pairwise sequence identity map, we first fragment the region of interest into 5 kb non-overlapping fragments. Then, we align every 5 kb fragment to every other 5 kb fragment using minimap2[5], retaining

only the best local alignment between each pair (**Methods**). As an example, we illustrate the alignment between two 5 kb fragments originating from the D8Z2 centromeric HOR array. Here, a target fragment$_i$ and a query fragment$_j$ are aligned to each other, generating multiple potential alignments. The highest-scoring alignment occurs between those with the most similar sequence and structure, and this guarantees registry among HORs wherever possible. Sequence identity is calculated from the highest-scoring alignment between each pair (**Methods**) and stored in an N × N matrix, while the others are discarded. Sequence identities between each pair are plotted as data points in R using ggplot2 such that the highest identities are dark red and the lowest identities are dark purple. Since the N × N matrix has identical information on each side of the diagonal, only one half of the matrix is presented.

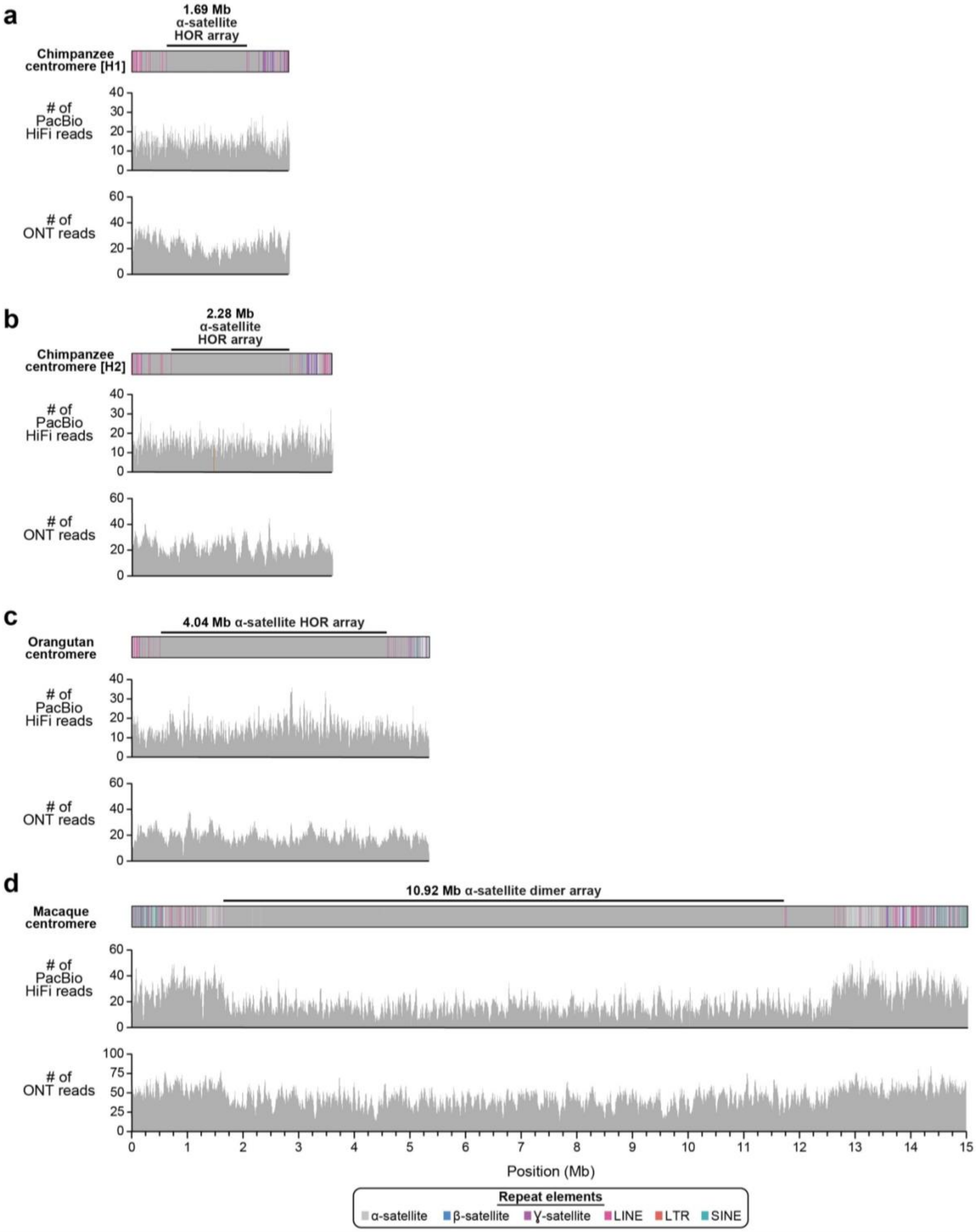

Supplementary Figure 4. Validation of the NHP centromeric regions with mapped long reads. a-d) Coverage of the **a)** chimpanzee (H1), **b)** chimpanzee (H2), **c)** orangutan, and **d)** macaque chromosome 8 centromeric regions with PacBio HiFi and ONT data generated from the same genome reveals largely uniform coverage. The increase in coverage on the edges of the macaque centromeric region is due to the inability to resolve the two haplotypes flanking the centromeric satellite array. Our results suggest that there are too few allelic differences to distinguish the flanking haplotypes. The macaque α-satellite dimer array, however, is fully resolved and does not show any signs of sequence collapse. All assemblies are to scale. H1, haplotype 1; H2, haplotype 2.

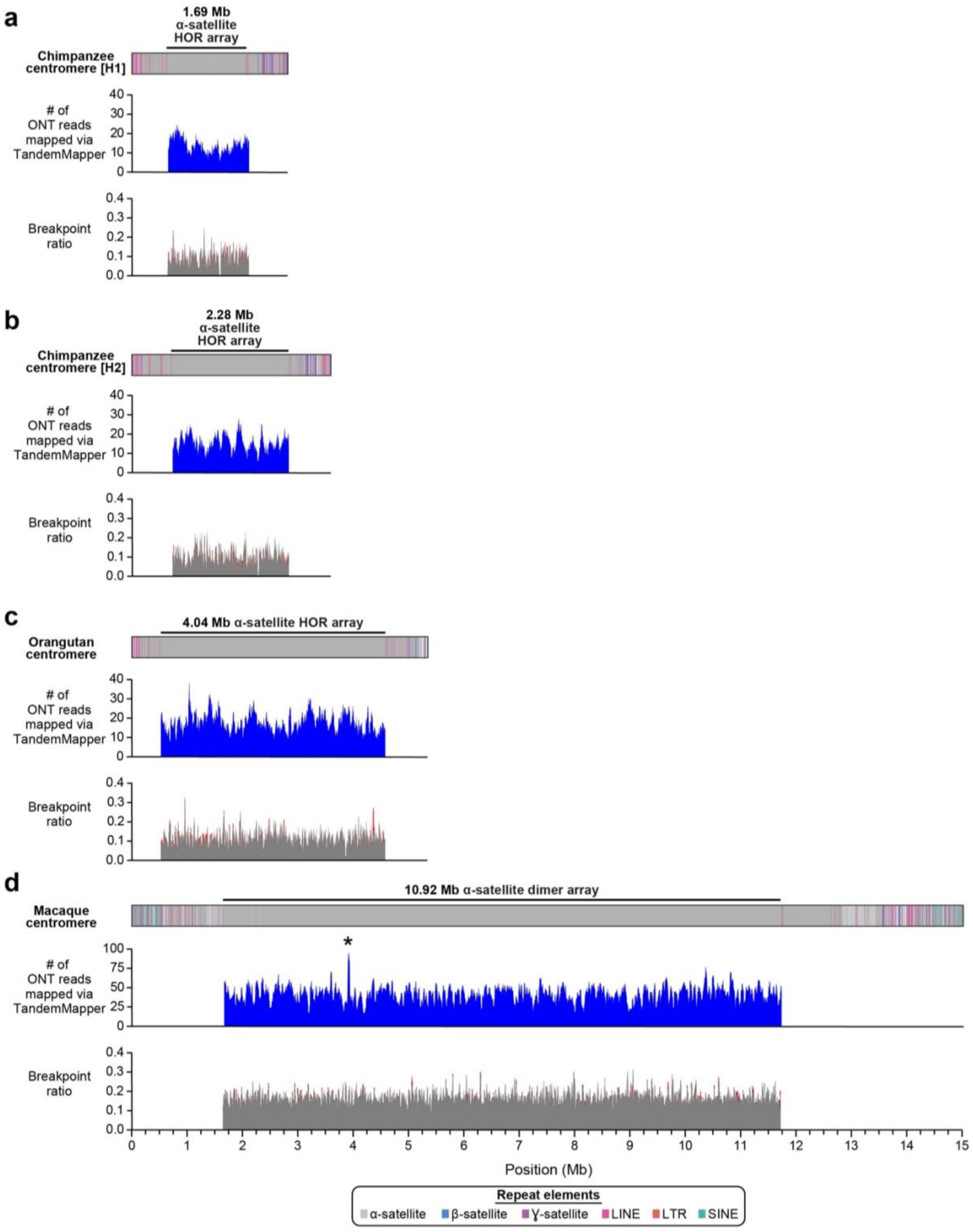

Supplementary Figure 5. Validation of the NHP centromeric regions with TandemQUAST analysis. a-d) ONT reads mapped with TandemMapper (top panel) and breakpoint ratios identified with TandemQUAST (bottom panel) for **a)** chimpanzee (H1), **b)** chimpanzee (H2), **c)** orangutan, and **d)** macaque chromosome 8 centromeric regions reveals a lack of large structural errors, except for a potential collapse identified in the macaque centromere (marked with an asterisk). All assemblies are to scale. H1, haplotype 1; H2, haplotype 2.

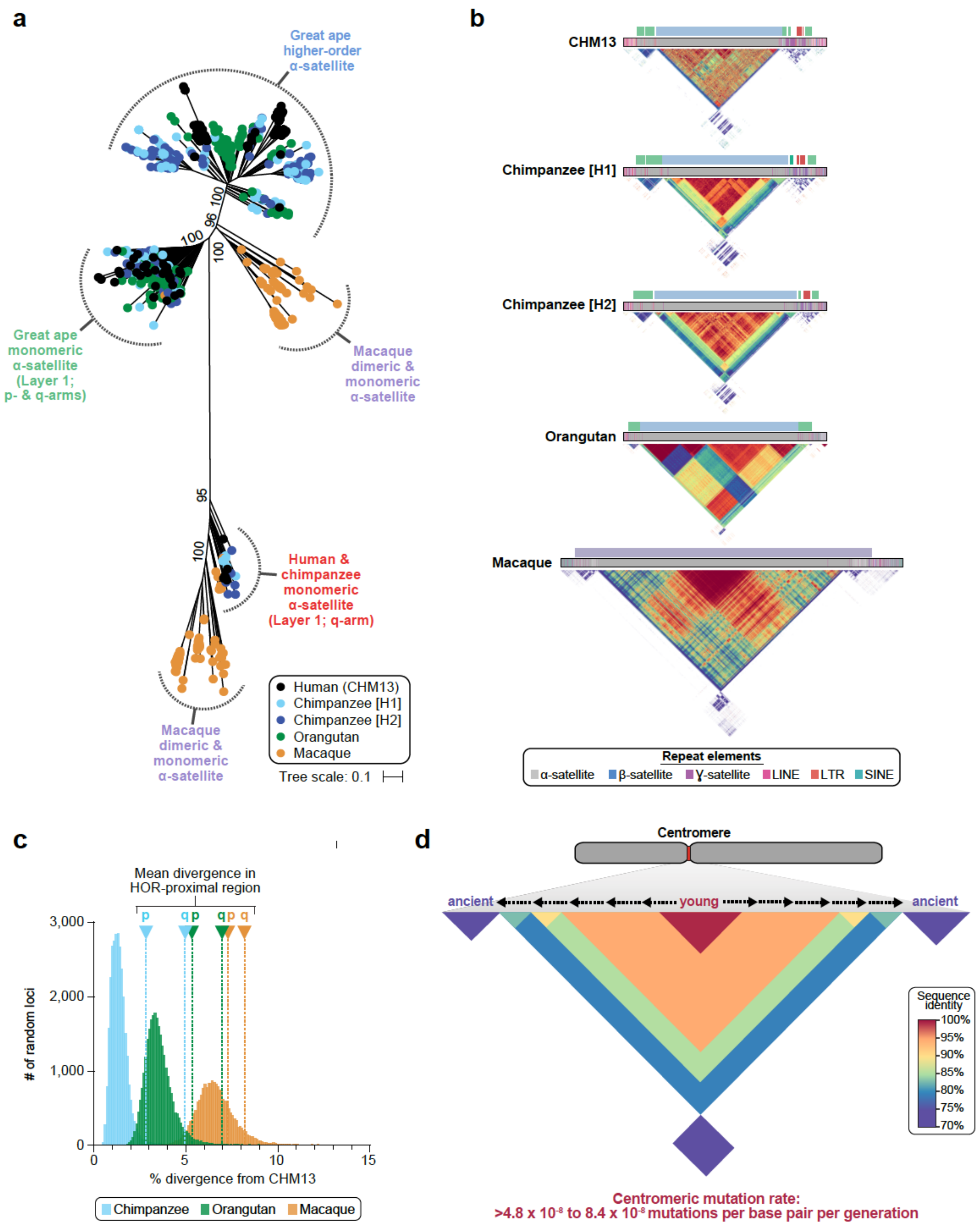

Supplementary Figure 6. Relative position of α-satellite phylogenetic clades. a) Phylogenetic tree with bootstrapping indicated at each major node. The clades are color coded, and the relative position

of the α-satellite in each clade is indicated in **Panel b**. **b)** Location of α-satellite from each clade from **Panel a**. The ancient monomeric α-satellite (red) is restricted to the q-arm in CHM13 and chimpanzee and represents the vestigial centromeric α-satellite from the common ancestor with macaque.
c) Histogram of the sequence divergence between CHM13 and chimpanzee, orangutan, or macaque at 19,926 random loci. **d)** Model of centromere evolution. Centromeric α-satellite HORs evolve in the center of the array via unequal crossing over and homogenization, pushing older, more ancient HORs to the edges, consistent with hypotheses previously put forth[6–8]. The centromeric mutation rate is estimated to be at least 4.8-8.4 x 10^{-8} mutations per base pair per generation, which is 2.2- to 3.8-fold higher than the mean basal mutation rate.

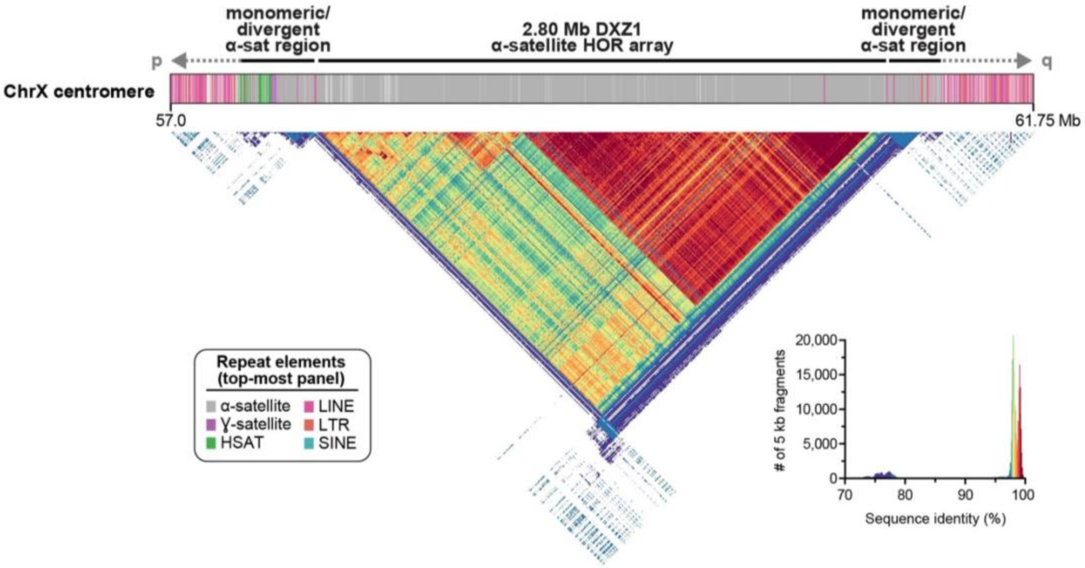

Supplementary Figure 7. Sequence composition and identity of the chromosome X centromeric region. Repeat structure and sequence identity map of the chromosome X centromeric region reveals >90% sequence identity across the HOR array and four distinct evolutionary layers that lack the symmetricity observed at the chromosome 8 HOR array, in concordance with prior analysis[9].

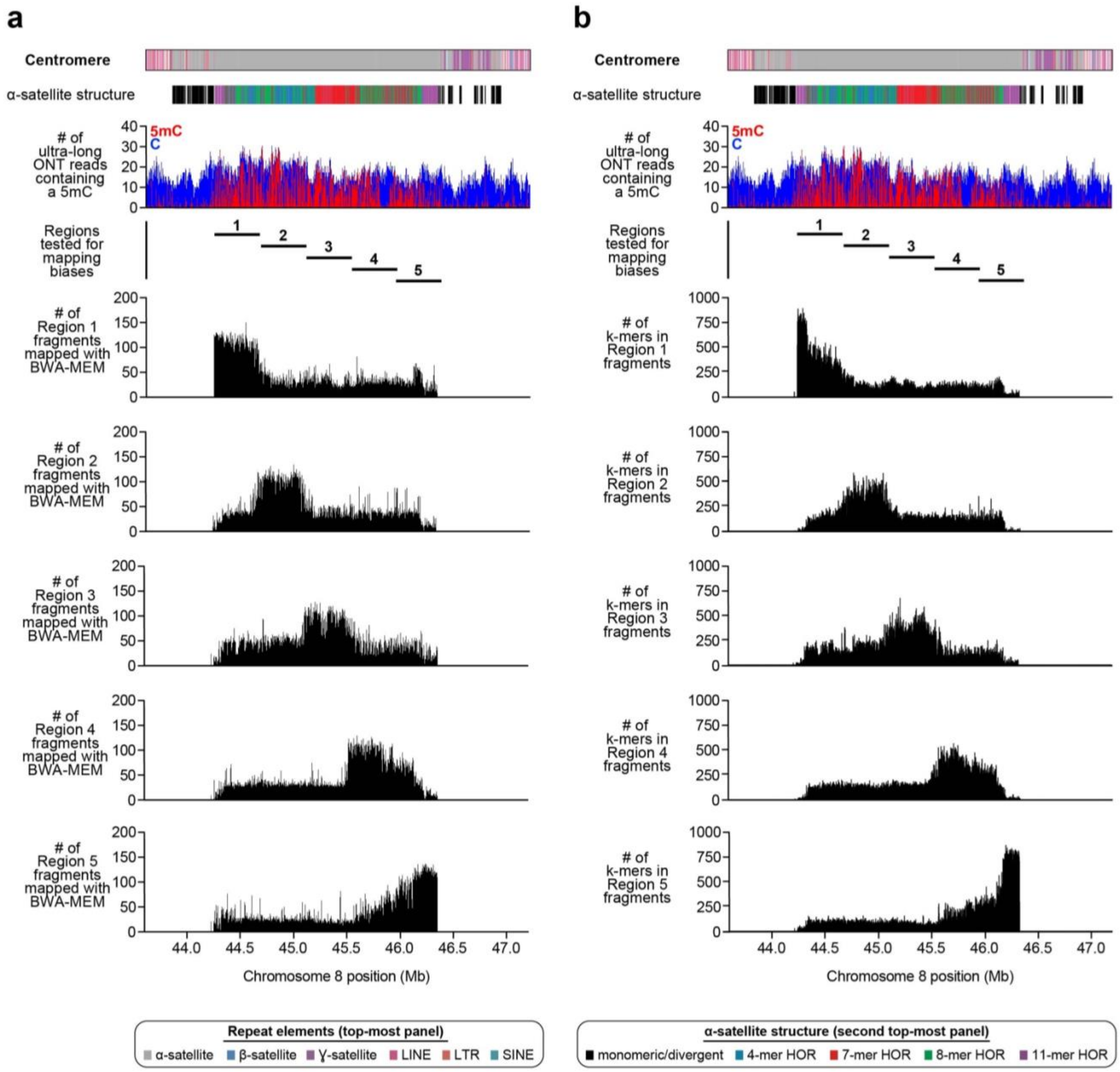

Supplementary Figure 8. Mappability of short reads within the D8Z2 α-satellite HOR array. To determine the mappability of short reads within the chromosome 8 centromeric HOR array, we performed a simulation where we generated 150 base fragments from five 416 kb regions across the HOR array and mapped them back to the D8Z2 α-satellite HOR array using **a**) BWA-MEM or **b**) our k-mer-based mapping approach (**Methods**). We find that the fragments mapped preferentially to the regions from which they originated. Importantly, we find that these fragments do not preferentially map to the hypomethylated region, where CENP-A is predicted to be located (**Fig. 2a**, **Extended Data Fig. 8**), indicating that mapping biases are not at an appreciable level in this region.

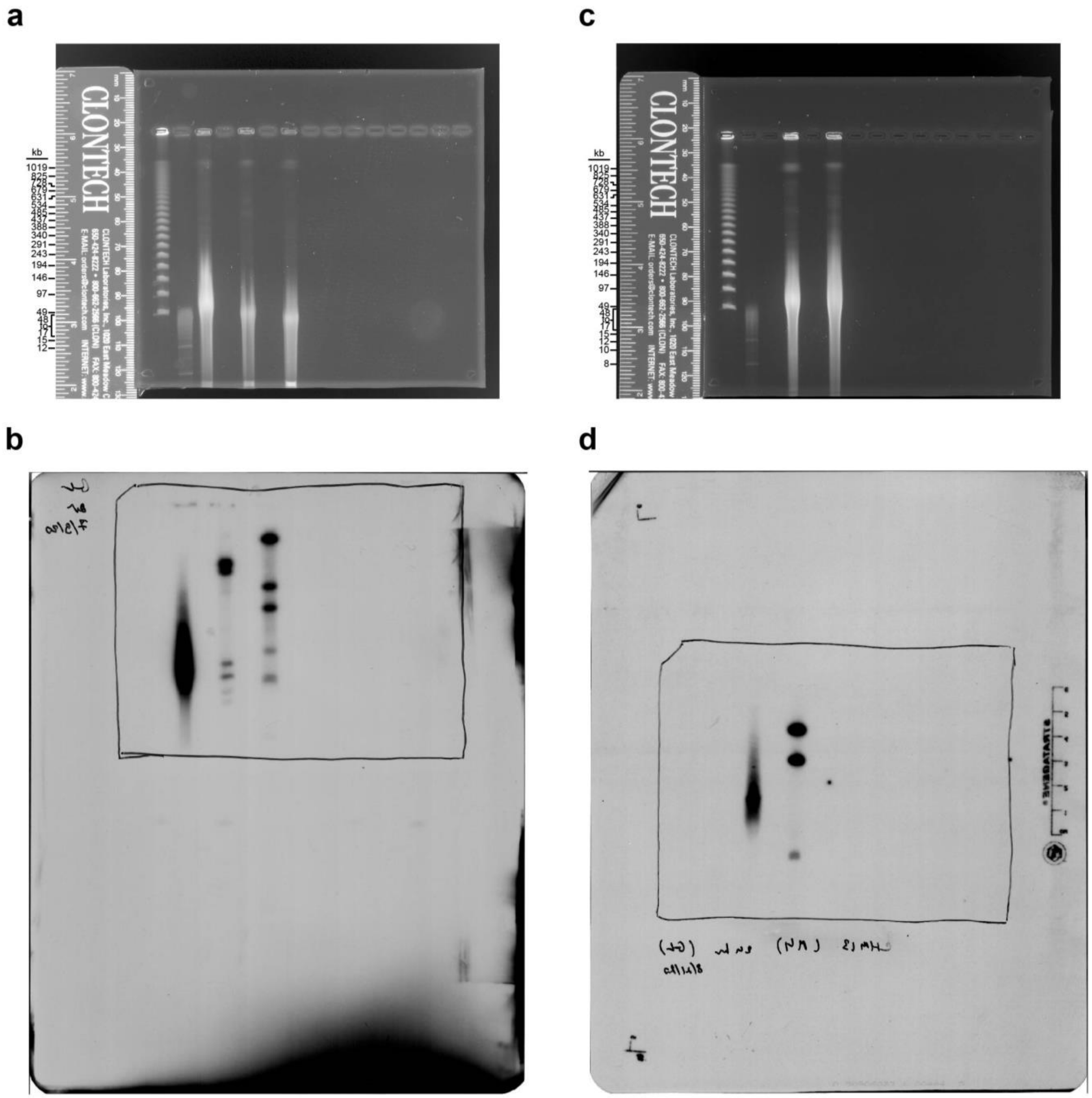

Supplementary Figure 9. Original, unprocessed images of pulsed-field gels (PFGs) and Southern blots. a-d) Raw, uncropped images of **a,c)** EtBr-stained PFGs and **b,d)** Southern blots used to assess the structure of the CHM13 **a,b)** chromosome 8 centromere (see **Fig. 2b** for cropped images) and **c,d)** chromosome 8q21.2 VNTR (see **Extended Data Figure. 1b** for cropped images). Molecular weight markers are labeled.

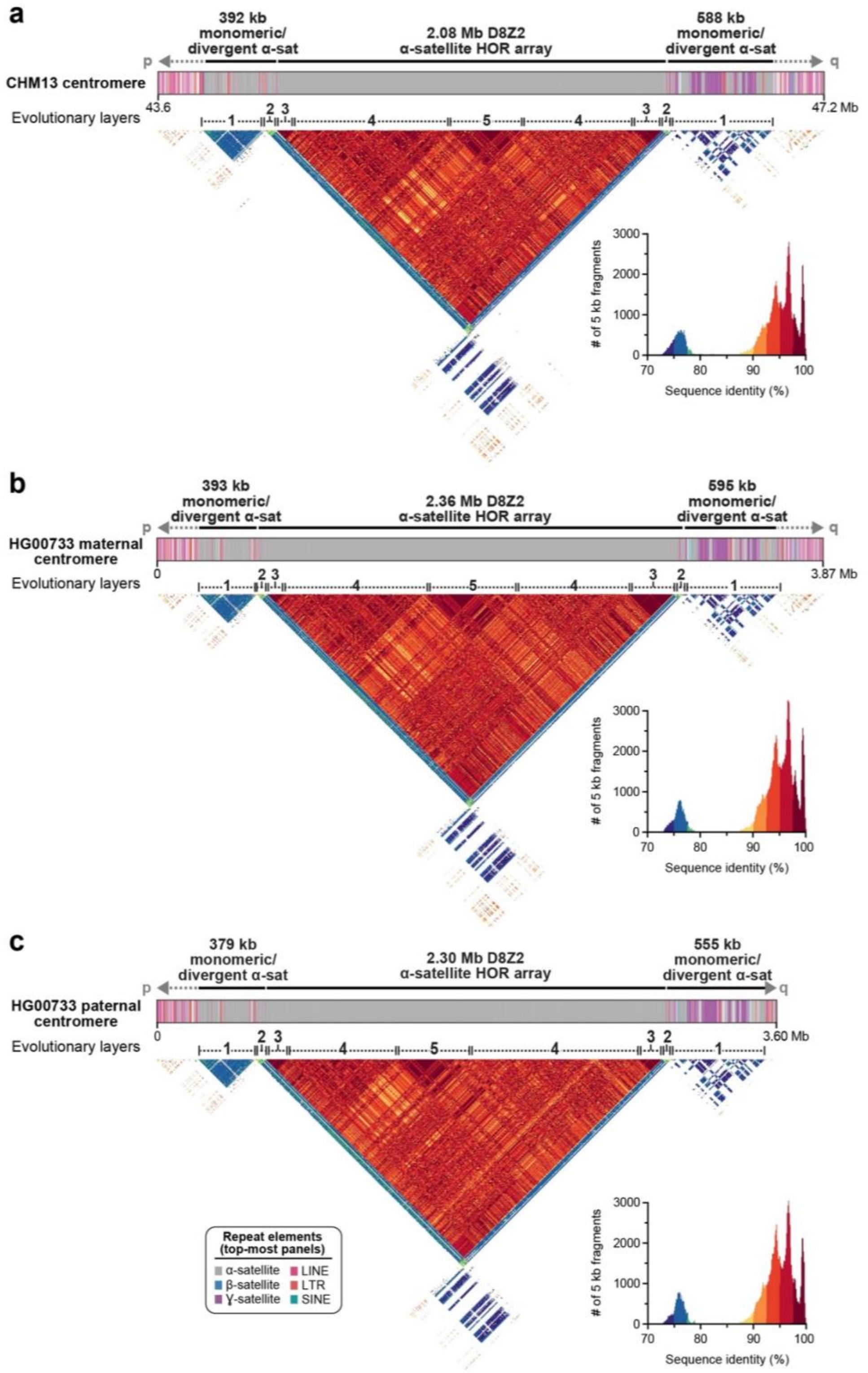

Supplementary Figure 10. Sequence identity heatmaps of human chromosome 8 centromeric regions, presented on the same color scale. a-c) Pairwise sequence identity heatmap of the **a)** CHM13, **b)** maternal HG00733, and **c)** paternal HG00733 chromosome 8 centromeric regions. All maps are shown on the same color scale and are consistent with those shown in **Supplementary Fig. 11**.

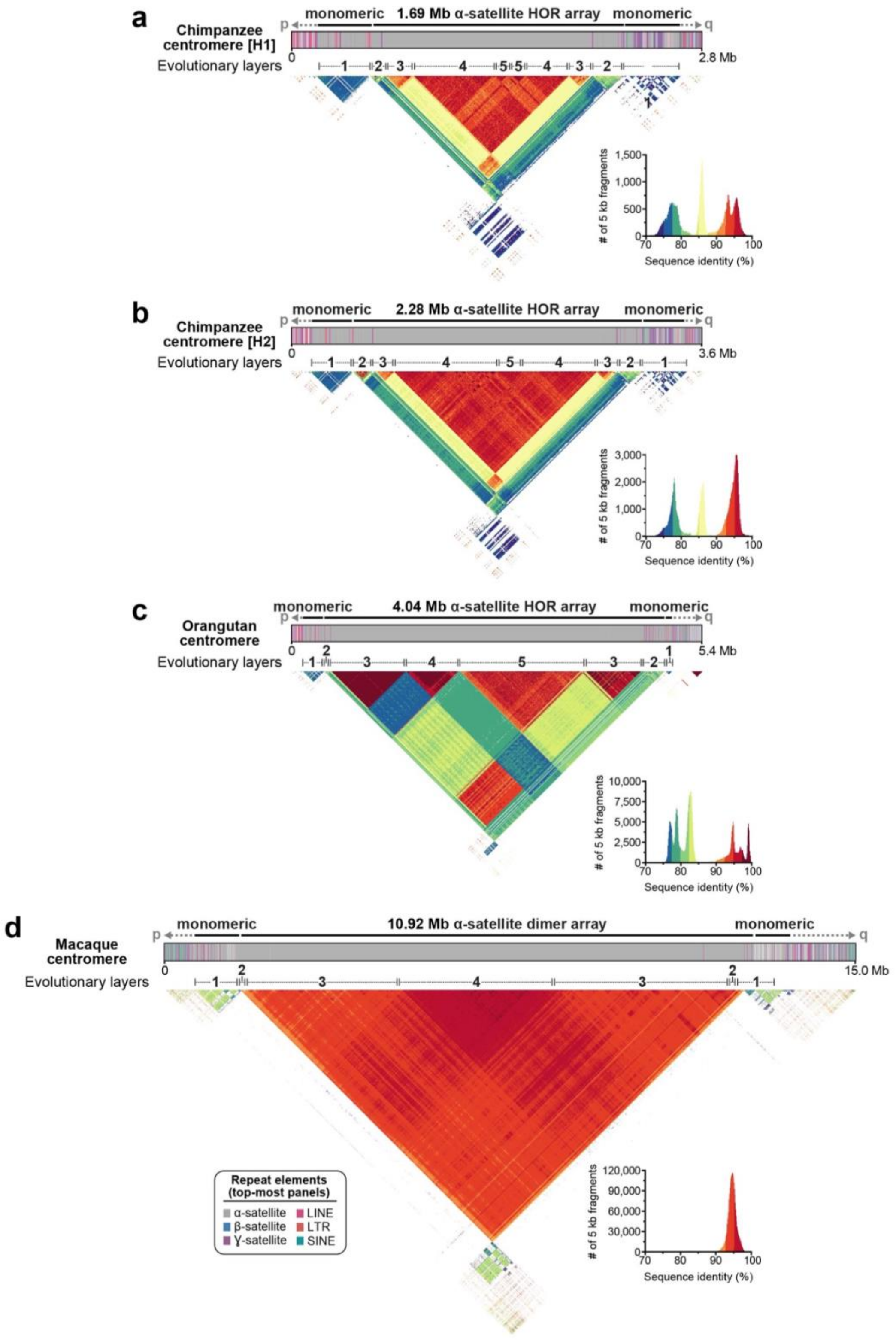

Supplementary Figure 11. Sequence identity heatmaps of nonhuman primate chromosome 8 centromeric regions, presented on the same color scale. a-d) Pairwise sequence identity heatmaps of the **a)** chimpanzee (H1), **b)** chimpanzee (H2), **c)** orangutan, and **d)** macaque chromosome 8 centromeric regions. All maps are shown on the same color scale and are consistent with those shown in **Supplementary Fig. 10**. H1, haplotype 1; H2, haplotype 2.

SUPPLEMENTARY TABLES

Supplementary Table 1. Genes with greater sequence identity to CHM13 chromosome 8 than GRCh38. See accompanying Excel file.

Supplementary Table 2. Differences in CHM13 and GRCh38 (hg38) chromosome 8 *DEFA* and *DEFB* genes. See accompanying Excel file.

Supplementary Table 3. Sequence and assembly of the HG00733 genome.

	Assembly*			PacBio HiFi data		ONT data	
**Species**	**Size (Gb)**	**No. of contigs**	**N50 (Mb)**	**Sequencing depth†**	**Read N50 (kb)**	**Sequencing depth†**	**Read N50 (kb)**
Human (HG00733)	6.08	1,592	34.89	33.48	13.5	94.0	32.7

*Assembled from PacBio HiFi data with hifiasm[10]

†Assumes a 3.1 Gb genome

Supplementary Table 4. Evolutionary layers within the CHM13 and HG00733 chromosome 8 centromeres.

Evolutionary layer	CHM13 chromosome 8 centromere (bp)			HG00733 maternal chromosome 8 centromere (bp)			HG00733 paternal chromosome 8 centromere (bp)		
	**p-arm**	**q-arm**	**Total**	**p-arm**	**q-arm**	**Total**	**p-arm**	**q-arm**	**Total**
**1**	323918	496831	820749	319969	496835	816804	319966	496757	816723
**2**	59301	57889	117190	59306	57925	117231	59301	57889	117190
**3**	92405	149484	241889	94781	261407	356188	122757	149640	272387
**4**	842106	577929	1420035	816758	780819	1597577	630628	974986	1605614
**5**	--	--	416216	--	--	408974	--	--	419009

Supplementary Table 5. Sequence and assembly of NHP genomes.

	Assembly*			PacBio HiFi data		ONT data	
**Species**	**Size (Gb)**	**No. of contigs**	**N50 (Mb)**	**Sequencing depth†**	**Read N50 (kb)**	**Sequencing depth†**	**Read N50 (kb)**
Chimpanzee (*Pan troglodytes*; Clint; S006007)	6.02	26,305	57.99	40.08	11.0	48.04	67
Orangutan (*Pongo abelii*; Susie; PR01109)	6.02	10,890	3.6	24.71	17.9	39.69	63.2
Macaque (*Macaca mulatta*; AG07107)	6.12	19,526	1.9	27.91	19.2	56.5	33.3

*Assembled from PacBio HiFi data with HiCanu[11]

†Assumes a 3.2 Gb genome for each species

Supplementary Table 6. CHM13 chromosome 8 centromeric mutation rate. See accompanying Excel file.

Supplementary Table 7. Heterozygous sites within CHM13 chromosome 8.

CHM13 chromosome 8 coordinate	Insertion size (bp)	% of ONT reads >50 kb long supporting the CHM13 chromosome 8 assembly	% of ONT reads >50 kb long supporting the alternate structure
chr8:21,025,201	8829-8923	58.33% (35/60)	41.67% (25/60)
chr8:80,044,843	7884	98.59% (70/71)	1.41% (1/71)
chr8:121,388,618	5928-6023	70% (42/60)	30% (18/60)

ONT: Oxford Nanopore Technologies

Supplementary Table 8. PacBio Iso-Seq datasets. See accompanying Excel file.

Supplementary Table 9. Datasets generated and/or used in this study. See accompanying Excel file.

Supplementary Table 10. CHM13 BACs used in this study. See accompanying Excel file.

SUPPLEMENTARY REFERENCES

1. Falconer, E. & Lansdorp, P. M. Strand-seq: a unifying tool for studies of chromosome segregation. *Semin. Cell Dev. Biol.* **24**, 643–652 (2013).
2. Sanders, A. D., Falconer, E., Hills, M., Spierings, D. C. J. & Lansdorp, P. M. Single-cell template strand sequencing by Strand-seq enables the characterization of individual homologs. *Nat Protoc* **12**, 1151–1176 (2017).
3. Porubský, D. *et al.* Direct chromosome-length haplotyping by single-cell sequencing. *Genome Res* **26**, 1565–1574 (2016).
4. Porubsky, D. *et al.* Fully phased human genome assembly without parental data using single-cell strand sequencing and long reads. *Nature Biotechnology* 1–7 (2020) doi:10.1038/s41587-020-0719-5.
5. Li, H. Minimap2: pairwise alignment for nucleotide sequences. *Bioinformatics* **34**, 3094–3100 (2018).
6. Smith, G. P. Evolution of repeated DNA sequences by unequal crossover. *Science* **191**, 528–535 (1976).
7. Shepelev, V. A., Alexandrov, A. A., Yurov, Y. B. & Alexandrov, I. A. The evolutionary origin of man can be traced in the layers of defunct ancestral alpha satellites flanking the active centromeres of human chromosomes. *PLOS Genetics* **5**, e1000641 (2009).
8. Salser, W. *et al.* Investigation of the organization of mammalian chromosomes at the DNA sequence level. *Fed. Proc.* **35**, 23–35 (1976).
9. Miga, K. H. Centromere studies in the era of ‘telomere-to-telomere’ genomics. *Experimental Cell Research* **394**, 112127 (2020).
10. Cheng, H., Concepcion, G. T., Feng, X., Zhang, H. & Li, H. Haplotype-resolved de novo assembly with phased assembly graphs. *arXiv:2008.01237 [q-bio]* (2020).
11. Nurk, S. *et al.* HiCanu: accurate assembly of segmental duplications, satellites, and allelic variants from high-fidelity long reads. *Genome Res.* gr.263566.120 (2020) doi:10.1101/gr.263566.120.
