# Supplementary material for: The structure, function and evolution of a complete human chromosome 8
Source: Nature. 2021 Apr 7;593(7857):101–7. doi: 10.1038/s41586-021-03420-7 (PMC8099727; doi:10.1038/s41586-021-03420-7)
Supplement: Supplementary file 2 — Reporting Summary [file 41586_2021_3420_MOESM2_ESM.pdf]

nature research

Corresponding author(s): Evan E. Eichler

Last updated by author(s): Feb 16, 2021

Reporting Summary

Nature Research wishes to improve the reproducibility of the work that we publish. This form provides structure for consistency and transparency in reporting. For further information on Nature Research policies, see our Editorial Policies and the Editorial Policy Checklist.

Statistics

For all statistical analyses, confirm that the following items are present in the figure legend, table legend, main text, or Methods section.

n/a	Confirmed	
☐	☒	The exact sample size (*n*) for each experimental group/condition, given as a discrete number and unit of measurement
☐	☒	A statement on whether measurements were taken from distinct samples or whether the same sample was measured repeatedly
☒	☐	The statistical test(s) used AND whether they are one- or two-sided<br>*Only common tests should be described solely by name; describe more complex techniques in the Methods section.*
☒	☐	A description of all covariates tested
☐	☒	A description of any assumptions or corrections, such as tests of normality and adjustment for multiple comparisons
☐	☒	A full description of the statistical parameters including central tendency (e.g. means) or other basic estimates (e.g. regression coefficient) AND variation (e.g. standard deviation) or associated estimates of uncertainty (e.g. confidence intervals)
☒	☐	For null hypothesis testing, the test statistic (e.g. *F*, *t*, *r*) with confidence intervals, effect sizes, degrees of freedom and *P* value noted<br>*Give P values as exact values whenever suitable.*
☒	☐	For Bayesian analysis, information on the choice of priors and Markov chain Monte Carlo settings
☒	☐	For hierarchical and complex designs, identification of the appropriate level for tests and full reporting of outcomes
☒	☐	Estimates of effect sizes (e.g. Cohen's *d*, Pearson's *r*), indicating how they were calculated

Our web collection on statistics for biologists contains articles on many of the points above.

Software and code

Policy information about availability of computer code

Data collection	Leica Application Suite X (v3.7), Oxford Nanopore Technologies MinKNOW (v2.0 - v19.12), and Pacific Bioscience Sequel II Instrument Control SW (v7.1 or v8.0).
Data analysis	Custom code for the SUNK-based assembly method is available at https://github.com/glogsdon1/sunk-based_assembly. Other software used in this study are publicly available and include Pacific Biosciences CCS algorithm (v3.4.1 or v4.0.0), HiCanu (v2.0), minimap2 (v2.17), Jellyfish (v2.2.4), pbmm2 (v1.1.0), Winnowmap (v1.0), Merqury (v1.1), BWA-MEM (v0.7.17), sambamba (v0.6.8), SAMtools (v1.9), BEDtools (v2.27.1), deepTools (v3.4.3), TandemTools (version available March 20th, 2020), StringDecomposer (version available February 28th, 2020), Nanopolish (v0.12.5), CHESS (v2.2), R (v1.1.383), Solve (v3.4), RepeatMasker (v4.1.0), ImageJ (v1.51), MAFFT (v7.453), mrsFAST (v3.4.1), Sickle (v1.33), and Cutadapt (v1.18).

For manuscripts utilizing custom algorithms or software that are central to the research but not yet described in published literature, software must be made available to editors and reviewers. We strongly encourage code deposition in a community repository (e.g. GitHub). See the Nature Research guidelines for submitting code & software for further information.

Data

Policy information about availability of data

All manuscripts must include a data availability statement. This statement should provide the following information, where applicable:

- Accession codes, unique identifiers, or web links for publicly available datasets
- A list of figures that have associated raw data
- A description of any restrictions on data availability

The complete CHM13 chromosome 8 sequence and all data generated and/or used in this study are publicly available and listed in Supplementary Table 9 with their BioProject, accession #, and/or URL. For convenience, we list their BioProjects and/or URLs here: complete CHM13 chromosome 8 sequence (PRJNA559484);

CHM13 ONT, Iso-Seq, and CENP-A ChIP-seq data (PRJNA559484); CHM13 Strand-Seq alignments (https://zenodo.org/record/3998125); HG00733 ONT data (PRJNA686388); HG00733 PacBio HiFi data (PRJEB36100); testis and fetal brain Iso-Seq data (PRJNA659539); and nonhuman primate [chimpanzee (Clint; S006007), orangutan (Susie; PR01109), and macaque (AG07107) ONT and PacBio HiFi data (PRJNA659034). All CHM13 BACs used in this study are listed in Supplementary Table 10 with their accession #s.

Field-specific reporting

Please select the one below that is the best fit for your research. If you are not sure, read the appropriate sections before making your selection.

☒ Life sciences ☐ Behavioural & social sciences ☐ Ecological, evolutionary & environmental sciences

For a reference copy of the document with all sections, see nature.com/documents/nr-reporting-summary-flat.pdf

Life sciences study design

All studies must disclose on these points even when the disclosure is negative.

Sample size	We generated a whole-chromosome assembly of human chromosome 8 and assembled the chromosome 8 centromere in a diploid human cell line and three diploid nonhuman primates in order to perform phylogenetic and comparative analyses. For phylogenetic tree reconstruction of the centromeric satellite, we used 150 data points from each genome, which resulted in a bootstrap value of 100 for all major branches of the tree (meaning, 100 out of 100 times, the same branch was observed in that clade when repeating the phylogenetic reconstruction on resampled data). For the centromeric mutation rate computation, we compared 1,002 10 kbp regions from across the chimpanzee, orangutan, and macaque genomes to the corresponding human region, which spans approximately 1.65 Mbp of sequence. This number of data points is the maximum number of points that can possibly be analyzed within this region (assuming 10 kbp windows) and is strengthened by the comparison across three different species (rather than just one). For gene copy number estimation, we analyzed 1,105 published high-coverage datasets spanning nine human superpopulations, which were all that were available for this analysis and provides a sufficiently high number of genomes to determine a median and standard deviation of gene copy number for each superpopulation with confidence. For droplet digital PCR (ddPCR), we performed seven technical replicates, which is four more than the standard three technical replicates used in such experiments. For the chromatin fiber-FISH, we generated three slides, which served as technical replicates, and identified multiple fibers showing the indicated CENP-A and methylation patterns. For the pulsed-field gel Southern blots, each experiment was performed twice with different restriction enzymes, and each result confirmed the expected banding pattern. For FISH on metaphase chromosome spreads, experiments were performed >3 times and generated several spreads with chromosome 8 FISH probes hybridized in the expected order. This number of FISH replicates meets or exceeds the standard number of experimental replication commonly accepted by the field.
Data exclusions	No data were excluded.
Replication	Computational experiments are deterministic and are, therefore, reproducible. Despite this expected reproducibility, computational experiments were run multiple times with different parameters to improve the experimental analysis. All attempts at replication were successful for both computation and wet-lab experiments.
Randomization	Randomization is not applicable to this study because we did not perform any experiments where there are treatment and control groups that would necessitate randomization between the subjects.
Blinding	Blinding is not applicable to this study because we did not perform any experiments where there are treatment and control groups that would necessitate blinding.

Reporting for specific materials, systems and methods

We require information from authors about some types of materials, experimental systems and methods used in many studies. Here, indicate whether each material, system or method listed is relevant to your study. If you are not sure if a list item applies to your research, read the appropriate section before selecting a response.

Materials & experimental systems

n/a	Involved in the study	
☐	☒	Antibodies
☐	☒	Eukaryotic cell lines
☒	☐	Palaeontology and archaeology
☒	☐	Animals and other organisms
☒	☐	Human research participants
☒	☐	Clinical data
☒	☐	Dual use research of concern

Methods

n/a	Involved in the study	
☐	☒	ChIP-seq
☒	☐	Flow cytometry
☒	☐	MRI-based neuroimaging

Antibodies

Antibodies used	Mouse monoclonal anti-CENP-A antibody (Enzo, ADI-KAM-CC006-E)

Antibodies used	Rabbit monoclonal anti-5-methylcytosine antibody (RevMAb, RM231)<br>Alexa Fluor 488 goat anti-rabbit (Thermo Fisher Scientific, A-11034)<br>Alexa Fluor 594 conjugated to goat anti-mouse (Thermo Fisher Scientific, A-11005)
Validation	The anti-CENP-A antibody was generated against a synthetic peptide consisting of aa3-19 of CENP-A, and mutation of this epitope in human cells prevents antibody binding (Logsdon et. al., JCB, 2015).<br><br>The anti-5-methylcytosine antibody was tested against 50, 5, and 0.5 ng of double stranded 5-hydroxymethylcytosine (5-hmC) DNA, 5-methylcytosine (5-mC) DNA, and unmethylated DNA on a dot blot, and it only detected the 5-mC DNA (see https://www.revmab.com/index.php/product/anti-5-methylcytosine-5-mc-rabbit-monoclonal-antibody-clone-rm231-5-mc/).

Eukaryotic cell lines

Policy information about cell lines

Cell line source(s)	CHM13hTERT (abbr. CHM13) cells were originally isolated from a hydatidiform mole at Magee-Womens Hospital (Pittsburgh, PA) as part of a research study (IRB MWH-20-054). Cryogenically frozen cells from this culture were grown and transformed using human telomerase reverse transcriptase (TERT) to immortalize the cell line. This cell line retains a 46,XX karyotype and complete homozygosity. Human HG00733 lymphoblastoid cells were originally obtained from a female Puerto Rican child, immortalized with the Epstein-Barr Virus (EBV), and stored at the Coriell Institute for Medical Research (Camden, NJ). Chimpanzee (Pan troglodytes; Clint; S006007) fibroblast cells were originally obtained from a male western chimpanzee named Clint (now deceased) at the Yerkes National Primate Research Center (Atlanta, GA) and immortalized with EBV. Orangutan (Pongo abelii; Susie; PR01109) fibroblast cells were originally obtained from a female Sumatran orangutan named Susie (now deceased) at the Gladys Porter Zoo (Brownsville, TX), immortalized with EBV, and stored at the Coriell Institute for Medical Research (Camden, NJ). Macaque (Macaca mulatta; AG07107) fibroblast cells were originally obtained from a female rhesus macaque of Indian origin and stored at the Coriell Institute for Medical Research (Camden, NJ).
Authentication	The CHM13hTERT cell line was authenticated via STR analysis and karyotyped to show a 46,XX karyotype (Miga et al., Nature, 2020). The other cell lines used in this study have not been authenticated to our knowledge.
Mycoplasma contamination	The CHM13hTERT cell line is negative for mycoplasma contamination (Miga et al., Nature, 2020). The other cell lines used in this study have not been assessed for mycoplasma contamination to our knowledge.
Commonly misidentified lines<br>(See ICLAC register)	No commonly misidentified cell lines were used in this study.

ChIP-seq

Data deposition

☒ Confirm that both raw and final processed data have been deposited in a public database such as GEO.

☒ Confirm that you have deposited or provided access to graph files (e.g. BED files) for the called peaks.

Data access links<br>*May remain private before publication.*	https://www.ncbi.nlm.nih.gov/sra/?term=SRR13278681<br>https://www.ncbi.nlm.nih.gov/sra/?term=SRR13278682<br>https://www.ncbi.nlm.nih.gov/sra/?term=SRR13278683<br>https://www.ncbi.nlm.nih.gov/sra/?term=SRR13278684
Files in database submission	CHM13_CA_ChIP_1_S3_R1_001.fastq.gz<br>CHM13_CA_ChIP_1_S3_R2_001.fastq.gz<br>CHM13_CA_ChIP_2_S4_R1_001.fastq.gz<br>CHM13_CA_ChIP_2_S4_R2_001.fastq.gz<br>CHM13_Input_1_S1_R1_001.fastq.gz<br>CHM13_Input_1_S1_R2_001.fastq.gz<br>CHM13_Input_2_S2_R1_001.fastq.gz<br>CHM13_Input_2_S2_R2_001.fastq.gz
Genome browser session<br>(e.g. UCSC)	Alignment of the CHM13 CENP-A ChIP-seq data to the CHM13 chromosome 8 assembly can be viewed on the UCSC Genome Browser session at the following link: https://genome.ucsc.edu/s/glogsdon1/CHM13_Chr8_CA_ChIP-seq.

Methodology

Replicates	Two independent replicates of CENP-A ChIP-seq (with chromatin input as a control) were performed on CHM13 cells and were in agreement with each other.
Sequencing depth	All samples were sequenced with 150 bp, paired-end Illumina sequencing, generating a total of 447,609,176 reads. The number of reads associated with each sample is listed below.<br><br>CHM13 CENP-A ChIP (Replicate 1) = 114,230,840 reads<br>CHM13 CENP-A ChIP (Replicate 2) = 131,316,036 reads<br>CHM13 Input (Replicate 1) = 98,173,458 reads<br>CHM13 Input (Replicate 2) = 103,888,842 reads

Antibodies	A mouse monoclonal anti-CENP-A antibody (Enzo, ADI-KAM-CC006-E) was used for the ChIP-seq experiments.
Peak calling parameters	All data were aligned to the CHM13 whole-genome assembly containing the contiguous chromosome 8 with the following BWA-MEM parameters: bwa mem -k 50 -c 1000000 {index} {read1.fastq.gz} {read2.fastq.gz}. The resulting SAM files were filtered using SAMtools with FLAG score 2308 to prevent multi-mapping of reads. With this filter, reads mapping to more than one location are randomly assigned a single mapping location, thereby preventing mapping biases in highly identical regions. The ChIP-seq data were downsampled to the same coverage across all datasets and normalized with deepTools bamCompare with the following parameters: bamCompare -b1 {ChIP.bam} -b2 {WGS.bam} --operation ratio --binSize 1000 -o {out.bw}. The resulting bigWig file was visualized on the UCSC Genome Browser using the CHM13 chromosome 8 assembly as an assembly hub.
Data quality	Data were quality-checked using FastQC (https://github.com/s-andrews/FastQC), and low-quality end bases were trimmed with Sickle (https://github.com/najoshi/sickle).
Software	deepTools bamCompare was used to compare the ratio of ChIP to Input reads aligning to the chromosome 8 centromere. The following parameters were used: bamCompare -b1 {ChIP.bam} -b2 {WGS.bam} --operation ratio --binSize 1000 -o {out.bw}.
